# Cryo-EM structure of haemoglobin at 3.2 Å determined with the Volta phase plate

**DOI:** 10.1038/ncomms16099

**Published:** 2017-06-30

**Authors:** Maryam Khoshouei, Mazdak Radjainia, Wolfgang Baumeister, Radostin Danev

**Affiliations:** 1 Department of Molecular Structural Biology, Max Planck Institute of Biochemistry, 82152 Martinsried, Germany; 2 The Clive and Vera Ramaciotti Centre for Cryo-EM, Department of Biochemistry and Molecular Biology, Monash University, Victoria, 3800 Melbourne, Australia

## Abstract

With the advent of direct electron detectors, the perspectives of cryo-electron microscopy (cryo-EM) have changed in a profound way. These cameras are superior to previous detectors in coping with the intrinsically low contrast and beam-induced motion of radiation-sensitive organic materials embedded in amorphous ice, and hence they have enabled the structure determination of many macromolecular assemblies to atomic or near-atomic resolution. Nevertheless, there are still limitations and one of them is the size of the target structure. Here, we report the use of a Volta phase plate in determining the structure of human haemoglobin (64 kDa) at 3.2 Å. Our results demonstrate that this method can be applied to complexes that are significantly smaller than those previously studied by conventional defocus-based approaches. Cryo-EM is now close to becoming a fast and cost-effective alternative to crystallography for high-resolution protein structure determination.

## Abstract (web summary)

Single particle cryo-EM is commonly used for the structure determination of large complexes. Here, the authors present the 3.2 Å resolution cryo-EM structure of human haemoglobin, which has a molecular weight of 64 kDa.

Cryo-electron microscopy (cryo-EM) has already established itself as a powerful tool for structure determination of protein complexes^1^. Thus far, protein complexes that have been successfully reconstructed to high resolution by single particle analysis (SPA) have molecular weights of ∼100 kDa or larger^2^. Given the radiation sensitivity of ice-embedded proteins, the low signal-to-noise ratio of cryo-EM images is a limitation for SPA^3^, restricting the size range of proteins that can be studied. In 1995, it was estimated, based solely on physical considerations, that the lower molecular weight limit of single particle cryo-EM would be 38 kDa^4^. It was suggested that the structure of 100 kDa proteins could be determined at 3 Å resolution from ∼10,000 particles. Later, it was proposed that the theoretical molecular weight limit might be as low as 17 kDa^5^. With the technology at that time it seemed that obtaining a 3 Å reconstruction would be reserved for complexes with a molecular weight upwards of 4 MDa^4^. Nowadays, obtaining ∼3 Å resolution reconstructions has become almost routine and has been achieved with complexes that are much smaller in size^1^. To date, the smallest protein solved to near-atomic resolution by single particle cryo-EM is the 3.8 Å resolution structure of the 93 kDa isocitrate dehydrogenase^2^. Even so, SPA reconstructions are still strongly biased towards larger symmetric complexes, indicating there is still a long way to go before the full potential of imaging proteins with electrons is reached.

The difficulties in routinely obtaining high-resolution reconstructions of small molecular weight proteins are predominantly due to poor representation of low spatial frequencies in electron micrographs obtained by conventional transmission electron microscopy (CTEM)^3^. CTEM utilizes phase contrast produced by spherical aberration (Cs) and the deliberate defocusing of the microscope’s objective lens. This approach creates oscillations in the contrast transfer function (CTF) of the microscope with some spatial frequencies of the object being transferred poorly, or not at all. One can compensate for this effect by varying the level of defocus from image to image that is typically in the range of several hundreds to thousands of nanometres. By combining images that have different levels of contrast for given spatial frequencies an accurate representation of an object can be obtained. Nevertheless, the limitations due to reduced signal-to-noise ratio resulting from contrast loss remain.

In-focus single particle cryo-EM enabled by the Volta phase plate (VPP) holds the promise of yielding up to a twofold boost in signal-to-noise ratio and therefore enhancing our ability to observe weak phase objects^6^. The signal-to-noise ratio of VPP images is high because transfer of contrast of low spatial frequencies is optimal and constant for images taken in focus. Unlike previous phase plate designs, VPP images also retain the high spatial frequencies of the specimen enabling structure determination at near-atomic resolution^7^,^8^,^9^,^10^. However, in-focus imaging with VPP requires very precise focusing^7^ and the typically strong Cs present in cryo-electron microscopes appears to be a limiting factor in attaining resolutions better than 3 Å by in-focus phase plate imaging^7^.

Enabled by the ability to estimate and correct the phase shift of the VPP in CTFFIND4 (ref. 11) and RELION-2 (ref. 12), we therefore used a hybrid approach combining the strengths of CTEM and VPP^10^. This involves applying a defocus of ∼500 nm and correcting for the effects of CTF^10^. We applied this strategy to tetrameric haemoglobin (Hgb) that mediates oxygen transport in blood and has a molecular weight of 64 kDa and C2 symmetry. We chose Hgb for its iconic status as the first protein structure alongside myoglobin that was solved using X-ray crystallography by Max Perutz in 1960, coincidently by overcoming the phase problem of X-ray crystallography^13^.

## Results

### Cryo-EM structure of haemoglobin

**Figure 1:**
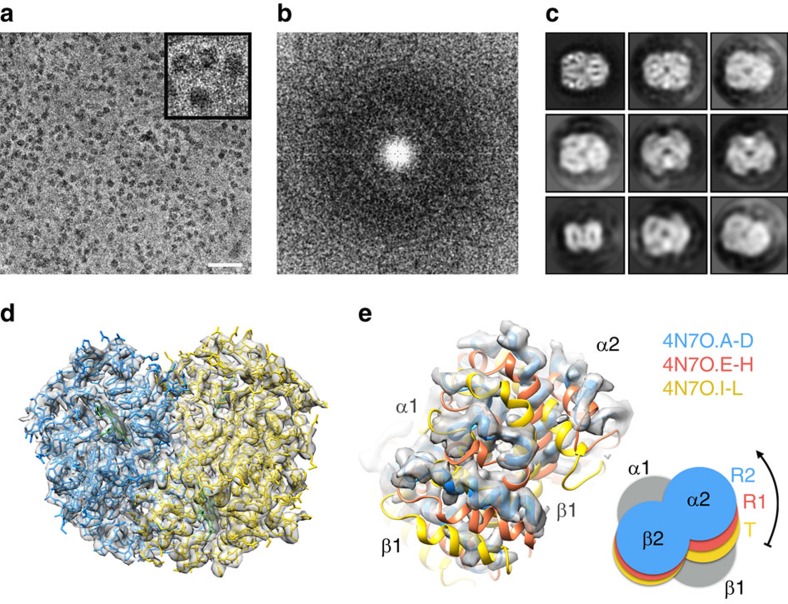
Phase plate imaging of 64 kDa Hgb. (**a**) Electron micrograph of metHgb recorded at ∼500 nm underfocus with the Volta phase plate (VPP) (scale bar, 30 nm). (**b**) Power spectrum of the image in **a**, featuring contrast transfer function (CTF) Thon rings permitting defocus and phase shift estimation. (**c**) The 2D class averages of Hgb showing secondary structure elements in projection. (**d**) Reconstructed 3D electron scattering potential map and model of Hgb. (**e**) VPP reconstruction fitted with three conformers of Hgb present in crystal structure PDB 4N7O. The reconstructed 3D map agrees best with chains A–D of PDB 4N7O representing the R2 state of Hgb.

**Figure 2:**
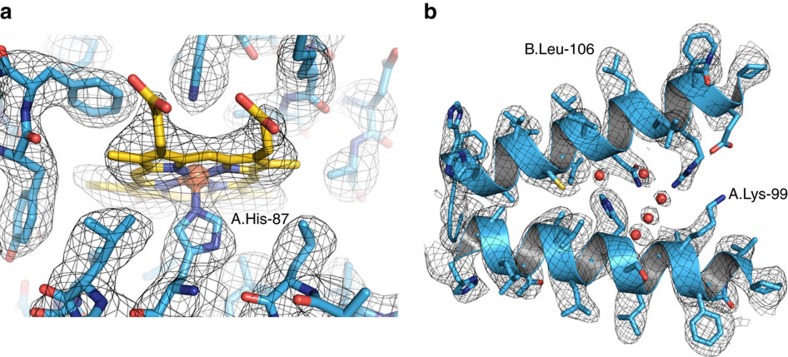
Hgb at 3.2 Å resolution. (**a**) The iron atom of the prosthetic haem group is coordinated by the proximal histidine residue, as evidenced by a strong density connecting them. (**b**) Side-chain details and putative water molecules in the 3D electron scattering potential map (red spheres).

**Figure 3:**
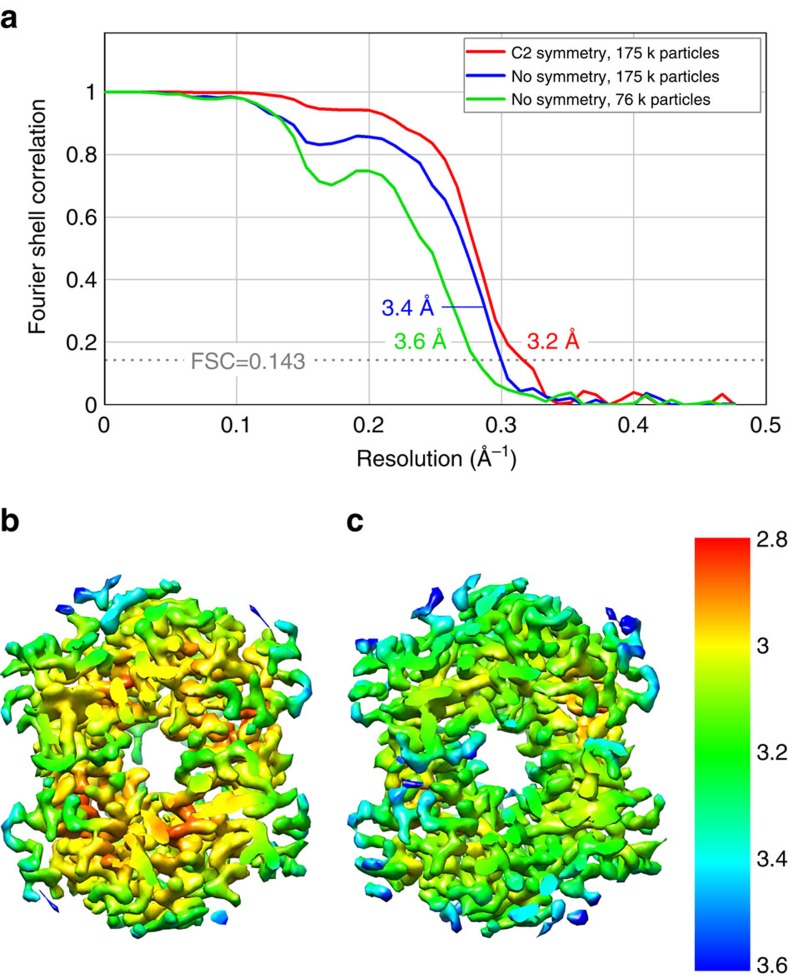
Resolution estimation. (**a**) Fourier shell correlation (FSC) plots indicating resolutions of 3.2 Å for the C2 symmetry (red line), 3.4 Å for the asymmetric (blue line) and 3.6 Å for the subset asymmetric (green line) reconstructions according to the FSC=0.143 criterion. (**b**) Local resolution estimation of the C2 symmetry map. (**c**) Local resolution estimation of the asymmetric map.

**Figure 4:**
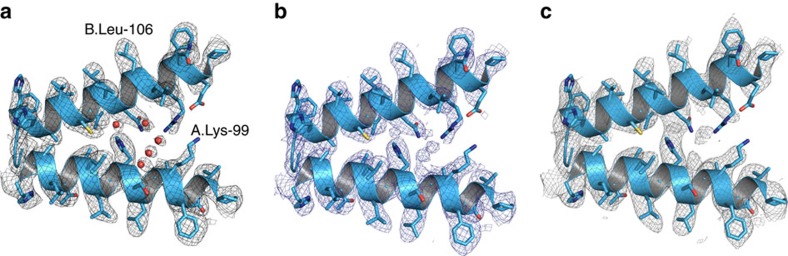
Comparison of 3D density maps. Representative side-chain densities in reconstructions with (**a**) imposed C2 symmetry, (**b**) no imposed symmetry and (**c**) no imposed symmetry using a subset of 76,150 particles.

Commercially sourced human Hgb is in the nonfunctional ferric (Fe^3+^) state referred to as metHgb. After vitrification of the metHgb, the sample was subjected to VPP-enabled imaging with multi-frame movies taken at low defocus, as described above. The movies were corrected for motion and radiation damage using MotionCor2 (ref. 14). Hgb particles were readily discernible in VPP images (Fig. 1a) and could be accurately picked because of their high contrast. Two-dimensional (2D) classification of automatically picked particles resulted in class averages with recognizable features and striking resemblance to the structure of Hgb (Fig. 1c). Class averages were selected for initial model building in EMAN2 (ref. 15) using the common-line technique and taking advantage of the C2 symmetry. RELION^16^ three-dimensional (3D) classification and refinement using half-split data sets of particles yielded the final map (Figs 1d and 2). The obtained 3D reconstruction had a resolution of 3.2 Å, as determined by the so-called ‘gold-standard’ Fourier shell correlation=0.143 criterion (Fig. 3). To evaluate the contribution of imposed symmetry in the result we performed an asymmetric reconstruction that produced a 3.4 Å map (Figs 3 and 4b). The relatively small resolution loss encouraged us to try an additional asymmetric reconstruction with less than half of the particles that generated a 3.6 Å map (Figs 3a and 4c).

At 3.2 Å resolution, side-chain densities and prosthetic haem groups are clearly resolved in the C2 symmetry map (Fig. 2). We used a molecular dynamics (MD)-based approach for model building and compared our atomic model with three conformers of ferrous (Fe^2+^) Hgb present in a single crystal (PDB-4N7O) adopting the tight (T) and two relaxed states (R1/R2)^17^. Rigid-body fitting was used to dock the α1 subunits yielding a good visual fit with a cross-correlation value of ∼69%. Superimposition of docked α1 subunits and corresponding tetramers yields cross-correlation values of 43, 47 and 62% for T, R1 and R2 states, respectively (Fig. 1e). This observation is in line with the fact that metHgb adopts an R-like state suggesting that conformational states can be determined for small proteins at high resolution without crystallization.

**Figure 5:**
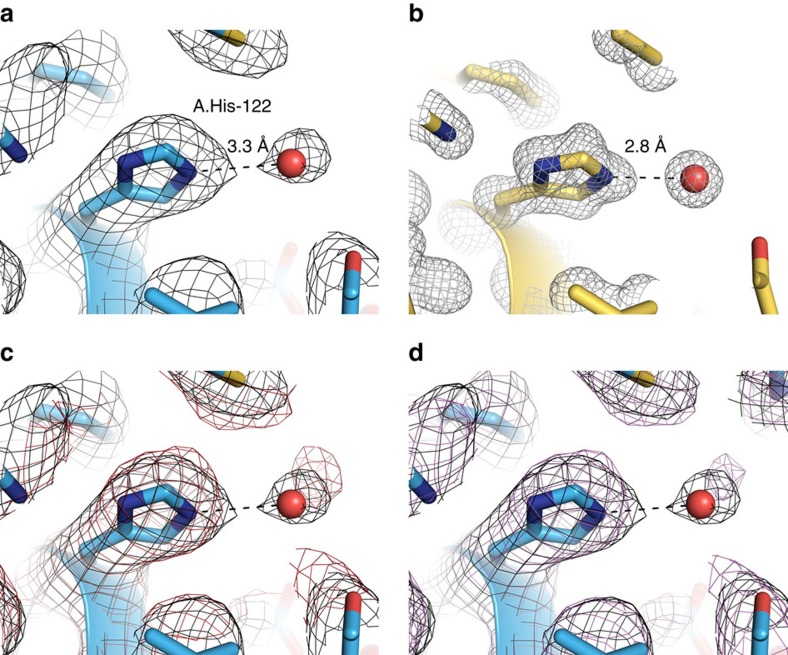
Validation of water molecules. (**a**) C2 symmetry reconstruction showing a water molecule density in the same location as observed in a crystal structure (**b**) (PDB-2DN1). (**c**,**d**) Same as (**a**) overlaid with two conjugate locations in the asymmetric reconstruction.

Close inspection of the 3D electron scattering potential map hinted at the presence of water molecule densities in our structure (Fig. 2b). The observed densities conform to small spherical shapes and are within hydrogen-bonding distances from hydrogen-bonding partners. They were consistent with positions of conserved water molecules in a high-resolution crystal structure^18^ (Fig. 5a,b). The putative water densities are also present at conjugate sites of the asymmetric map that further supports their fidelity (Figs 4b and 5c,d).

## Discussion

Our results showcase how cryo-EM can be used to determine the predominant conformational state of a protein in solution. It has become increasingly clear that allosteric models based on states arrested by tight crystal contacts potentially fail to provide a complete structure/function portrait and may be divergent from solution studies^17^. SPA is inherently better suited than crystallography for visualizing the full spectrum of conformational states that proteins adopt^19^. Obtaining high-resolution structures of solution states may indeed be one of the main applications of structure determination by VPP as a technique complementary to X-ray crystallography and nuclear magnetic resonance spectroscopy.

Based on the results presented here, we expect that the VPP will help in structure determination of proteins below 100 kDa. In conjunction with improved automation, and next-generation direct electron detectors, the range of samples accessible by cryo-EM will continue to grow.

## Methods

### Sample preparation

Human Hgb was commercially sourced (Sigma-Aldrich, St Louis, MO, USA) and dissolved in 50 mM Tris buffer at pH 7.6 to a final concentration of 1.5 mg ml^−1^. Frozen-hydrated specimens were prepared on plasma-cleaned Quantifoil R1.2/1.3 holey carbon EM grids (Quantifoil, Großlöbichau, Germany) using a Vitrobot Mark III (FEI, Hillsboro, OR, USA) 5 s blotting time, 85% humidity and −5 mm blotting offset.

### Data acquisition

**Figure 6:**
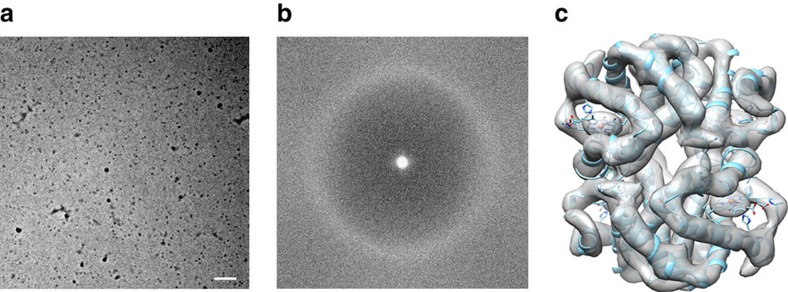
In-focus phase plate imaging of 64 kDa Hgb. (**a**) Electron micrograph of metHgb recorded at ∼20 nm underfocus with the Volta phase plate (VPP) (scale bar, 50 nm). (**b**) Power spectrum of the image in **a**, featuring continuous signal without CTF oscillations across the frequency spectrum and visibility of the amorphous ice ring at 3.7 Å. (**c**) Reconstructed 3D electron scattering potential map and fitted model of Hgb from crystal structure PDB-1A9W.

Automated data collection was performed on a Titan Krios electron microscope (FEI) operated at 300 kV and equipped with a K2 Summit direct detector, a Quantum energy filter (Gatan, Pleasanton, CA) and an FEI Volta phase plate (FEI) using SerialEM^20^ software. Movies comprising 40 frames, 2 s exposure time and a total dose of 40 e^−^ Å^−2^ were recorded on a K2 Summit direct detection camera (Gatan) in counting mode, at a calibrated magnification of 95,200 corresponding to a magnified pixel size of 0.525 Å. The small pixel size was selected to get better signal-to-noise ratio in the high-resolution region by placing it below the half-Nyquist frequency of the detector where the detective quantum efficiency is higher. For comparison, in our first attempt at solving the structure of Hgb we used a pixel size of 1.35 Å with in-focus VPP data collection and a set of ∼10,300 particles from 233 micrographs yielded a 6 Å density map (Fig. 6). The second attempt presented here comprised 2,261 micrographs acquired in one 89 h microscope session.

### Data processing

The recorded movies were subjected to motion correction with MotionCor2 (ref. 14). Following CTF estimation with CTFFIND4 (ref. 11), 705 micrographs with measured resolutions worse than 4 Å were excluded that left 1,556 micrographs for further processing. Particles were picked with Gautomatch (developed by Dr Kai Zhang, MRC Laboratory of Molecular Biology, Cambridge, UK, http://www.mrc-lmb.cam.ac.uk/kzhang/Gautomatch/). Subsequently, 283,600 particles were extracted in RELION-2 (ref. 12) using a box size of 100 pixels. After performing 2D classification in RELION-2, the best-looking 2D class averages, as judged by visual inspection, were selected to build an initial model in EMAN2 (ref. 15) using the common-line approach. After two rounds of 3D classification with 2 classes each, 175,374 particles from the higher-resolution class were subjected to 3D auto-refinement in RELION-2. The final map was sharpened with a measured B-factor of −176 Å^2^. Local resolution was calculated with *blocres* from the Bsoft^21^ package. For the asymmetric reconstruction, 3D auto-refinement in RELION-2 was repeated using the same set of 175,374 particles without imposing symmetry. Another asymmetric reconstruction was performed from a subset of 76,150 particles that were selected by an additional round of 3D classification using 5 classes and no image alignment. The full and subset asymmetric reconstructions were sharpened with measured B-factors of −179 and −157 Å^2^ respectively. It must be noted that the 3D classification and refinement calculations for the Hgb data set were more than 10 times slower than those for similarly sized data sets of larger particles. This was due to the low amount of signal in the Hgb particles that produced much broader Bayesian probability distributions and in turn caused wider searches in RELION. Flexible fitting of the Hgb crystal structure was performed using the NAMD routine in MDFF^22^ followed by rebuilding with COOT^23^, real-space refinement in PHENIX^24^ and final refinement with REFMAC^25^ with half-map cross-validation^26^. The data collection, refinement parameters and model statistics are summarized in Table 1.

### Data availability

The C2 symmetry cryo-EM map and the refined atomic coordinates of Hgb were deposited to the Electron Microscopy Data Bank (EMDB) and Protein Data Bank (PDB) with accession codes EMD-3488 and PDB-5NI1, respectively. The full and subset asymmetric cryo-EM maps were deposited to the EMDB with accession codes EMD-3650 and EMD-3651, respectively. Raw data were made available at the Electron Microscopy Pilot Image Archive (EMPIAR) with accession code EMPIAR-10084. Other data that support the findings of this study are available from the corresponding author on reasonable request.

## Additional information

**How to cite this article:** Khoshouei, M. *et al*. Cryo-EM structure of haemoglobin at 3.2 Å determined with the Volta phase plate. *Nat. Commun.* **8,** 16099 doi: 10.1038/ncomms16099 (2017).

**Publisher’s note**: Springer Nature remains neutral with regard to jurisdictional claims in published maps and institutional affiliations.

## Figures and Tables

**Table 1:** Data collection, refinement parameters and model statistics.

*Data collection*	
Particles used in final 3D refinement	175,374
Pixel size (Å)	0.525
Defocus (μm)	−0.5
Voltage (kV)	300
Electron dose (e^−^Å^−2^)	40
	
*R.m.s. deviations*	
Bonds (Å)	0.01
Angles (°)	1.53
	
*Validation*	
Clashcore, all atoms	3.53
Good outliers (%)	0.0
	
*Ramachandran plot*	
Favoured (%)	95.23
Allowed (%)	4.77
Outliers (%)	0.0
	
*Refinement of C2 symmetry map (175,374 particles)*	
Resolution (Å)	3.2
Map sharpening B-factor (Å^2^)	−176
Fourier shell correlation criterion	0.143
	
*Refinement of asymmetric map (175,374 particles)*	
Resolution (Å)	3.4
Map sharpening B-factor (Å^2^)	−179
Fourier shell correlation criterion	0.143
	
*Refinement of asymmetric map (76,150 particles)*	
Resolution (Å)	3.6
Map sharpening B-factor (Å^2^)	−157
Fourier shell correlation criterion	0.143
